# Integrated Behavioral Health Services and Psychosocial Symptoms in Children

**DOI:** 10.1001/jamanetworkopen.2025.32020

**Published:** 2025-09-16

**Authors:** Jihye Kim, Megan B. Cole, Jessica Rosenberg, Anita Morris, Emily Feinberg, R. Christopher Sheldrick

**Affiliations:** 1 Research Support Services Department, Boston Medical Center, Boston, Massachusetts; 2 Department of Health Law, Policy, & Management, Boston University School of Public Health, Boston, Massachusetts; 3 Hassenfeld Child Health Innovation Institute, Brown University School of Public Health, Providence, Rhode Island; 4 Department of Psychiatry, University of Massachusetts Chan Medical School, Worcester

## Abstract

**Importance:**

Studies evaluating integrated pediatric behavioral health care using electronic medical record data are limited.

**Objective:**

To evaluate the association of receipt of integrated behavioral health services with changes in psychosocial symptoms among children receiving care at federally qualified health centers with behavioral health integration.

**Design, Setting, and Participants:**

This cohort study used electronic medical record data (June 2020 to April 2023) from children aged 4 to 18 years with an identified behavioral health concern at 4 Massachusetts federally qualified health centers with integrated behavioral health care. Data were analyzed in October 2024.

**Exposures:**

Receipt of integrated behavioral health services, categorized into 3 treatment groups: (1) receipt of behavioral health clinician (BHC) encounters, (2) community health worker encounters, and (3) psychotropic prescriptions. The control group included similar children who did not receive any of these treatments.

**Main Outcomes and Measures:**

The primary outcome was psychosocial symptom score based on the 17-item Pediatric Symptom Checklist (PSC-17). After applying propensity scores to match children receiving treatment and control groups on their baseline characteristics, associations of receiving a treatment with psychosocial symptoms were estimated by comparing scores before vs after treatment using linear regression models.

**Results:**

Of 942 unique children, 542 (57.5%) received any type of treatment and 400 (42.5%) were in the control group. Children with a BHC encounter and children without any treatment had similar baseline characteristics (female sex: 206 children [58.7%] vs 204 children [56.7%]; mean [SD] age, 11.8 [3.5] vs 11.7 [3.4] years). After having at least 1 encounter with a BHC, PSC-17 scores among children in the treatment group were 1.51 (95% CI, −2.65 to −0.37) points lower compared with the control group. After receiving a psychotropic prescription, PSC-17 scores among children in the treatment group were 2.21 (95% CI, −3.89 to −0.54) points lower compared with the control group. No statistically significant changes were observed among children with at least 1 community health worker encounter (−0.53 points; 95% CI, −1.86 to 0.80 points).

**Conclusions and Relevance:**

In this cohort study of children at federally qualified health centers implementing behavioral health integration, receipt of encounters with BHCs and psychotropic prescriptions were associated with improved psychosocial symptoms, suggesting that expanding integrated pediatric behavioral health care might enhance behavioral health outcomes among marginalized pediatric populations.

## Key Points

**Question:**

Are integrated pediatric behavioral health services associated with improved psychosocial symptoms in children who receive care at federally qualified health centers?

**Findings:**

This cohort study included 942 unique children aged 4 to 18 years with an identified behavioral health concern at 4 Massachusetts federally qualified health centers with integrated behavioral health care. Receipt of encounters with behavioral health clinicians and psychotropic prescriptions were associated with improved psychosocial symptoms, although there was no association of community health worker encounters with changes in symptoms.

**Meaning:**

These findings suggest that integrated behavioral health treatment at federally qualified health centers may improve psychosocial symptoms among children identified with behavioral health concerns.

## Teaser

This cohort study evaluates associations of receipt of integrated behavioral health services with changes in psychosocial symptoms among children receiving care at federally qualified health centers.

## Introduction

Integrated behavioral health (BH) services in pediatric primary care are increasingly recommended to address insufficient access to quality BH care.^1,2,3^ The rational is clear—pediatric primary care is associated with high engagement and low stigma as compared with specialty care^4^; thus, it is perceived as an ideal setting to improve access to BH care. Most models of integrated care incorporate evidence-based treatments, such as psychotherapy and psychotropic medications, which have been tested in randomized clinical trials (RCTs) and found to improve outcomes.^5,6,7,8^ In short, integrated BH care models are hypothesized to work because they improve access to effective treatments.

A growing literature provides direct evidence of the effectiveness of integrated BH care for improving accessibility, family engagement, and patient outcomes.^5,6,7,8^ Despite its methodological rigor, the generalizability of this RCT evidence to clinical practice conditions can be questioned.^9^ For example, although there are studies of broad-based models that address the full spectrum of disorders seen in primary care,^10^ many RCTs in research settings strictly follow preplanned treatment procedures that focus on a single focal problem.^11,12^ Such experiments often include relatively homogeneous groups of children who were selected for treatment based on certain inclusion and exclusion criteria, which may not be generalizable to the cases of actual clinical practice where children often have more complex conditions.^12^

Thus, research on the impact of integrated care models also benefits from a second kind of evidence—that is, indirect evidence that supports the “minimal, sequential clinical assumptions that must be verified using empirical evidence.”^13^ As described previously, minimal clinical assumptions for the impact of BH care are (1) that integrated BH care models improve access to BH care and (2) that access to BH care is associated with improved outcomes. By evaluating these assumptions, analyses of evidence regarding the delivery and/or impact of medical services derived from sources other than traditional clinical trials,^9,14^ such as electronic medical records (EMRs), can play a valuable role in the evaluation of clinical care.^12,15^

Research on the integrated BH model known as Transforming and Expanding Access to Mental Health Universally in Pediatrics (TEAM UP) provides a useful example.^16^ TEAM UP was codeveloped with federally qualified health centers (FQHCs), community-based centers that provide comprehensive, culturally responsive primary care services to economically and socially marginalized populations that lack access to high-quality care.^17^ The TEAM UP integrated BH care pathway (Figure) includes screening for BH needs, identification of BH concerns, and connection to care. Care may involve visits with integrated BH clinicians (BHCs) who provide evidence-based BH services, encounters with community health workers (CHWs) who support families by navigating systems and addressing health-related social needs,^18,19^ and/or follow-up from primary care practitioners (PCPs) who can provide medication treatment.^19^ Prior analyses of medical claims data found associations of TEAM UP with use of BH care; children’s engagement with PCPs for BH concerns and receipt of BH services (Figure) increased at practices that implemented TEAM UP compared with practices that did not implement TEAM UP.^20,21^ However, evidence of associations with improved health outcomes has been limited to a small descriptive cohort study, which found that children who received services through TEAM UP practices experienced lower BH symptoms over time and better school functioning.^22^ Thus, additional evidence is needed to support the association of TEAM UP integrated services with health outcomes.

**Figure.**
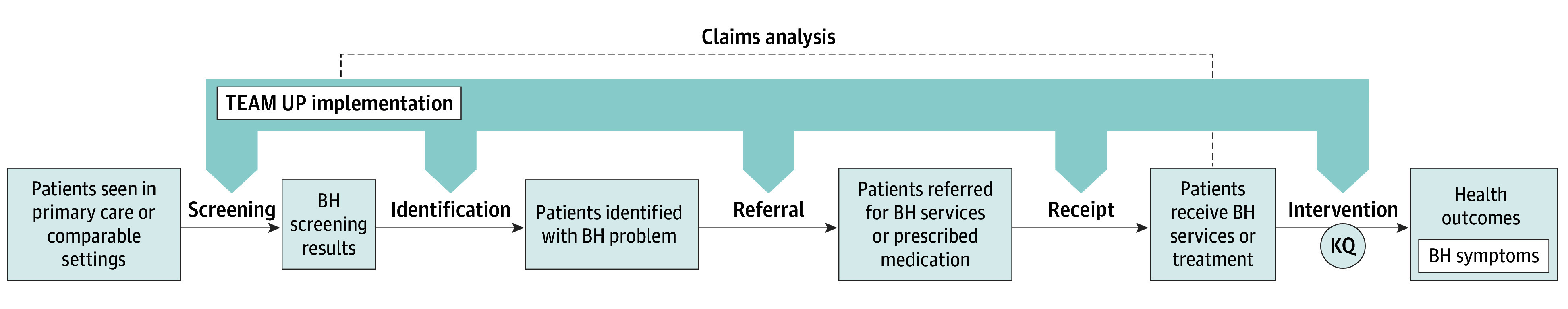
Transforming and Expanding Access to Mental Health Universally in Pediatrics (TEAM UP) Analytic Framework: Evaluating Integrated Behavioral Health (BH) Services The TEAM UP initiative is intended to strengthen pediatric primary care by integrating evidence-based BH services. It emphasizes early identification and prevention of BH concerns while improving access to necessary treatment. Services are delivered by an integrated care team of BH clinicians (BHCs), community health workers (CHWs), and primary care practitioners (PCPs), who work together to ensure timely and effective care. Licensed BHCs conduct assessments, provide diagnoses, offer consultations, and implement interventions for BH conditions. CHWs, tailored to the shared characteristics of patient population, serve in each practice (eg, language and cultural background), provide education, coordinate care to address gaps in services, and assess medical, behavioral, and health-related social needs. PCPs provide preventive and acute or chronic care, make specialty referrals, and prescribe and manage first line medications for common BH conditions. KQ indicates key question.

To address this evidence gap, we used propensity-matched pre-post regression analyses to compare changes in screening scores among children who received specific integrated BH services to matched controls who did not receive these services. To do so, we analyzed EMR data from 4 FQHCs that implemented TEAM UP.

## Methods

### Data Sources and Study Setting

This cohort study was approved by the Boston Medical Center institutional review board and followed the Strengthening the Reporting of Observational Studies in Epidemiology (STROBE) reporting guideline. Because data were deidentified, informed consent was waived. EMR data from June 2020 through April 2023 were extracted from 4 Massachusetts-based FQHCs that implemented TEAM UP starting in 2019. Each month, FQHCs submitted deidentified EMR data that included information on patient demographics, detection of BH concerns, receipt of primary care and integrated BH services, prescription of psychotropic medications, BH screening data, and structured fields from the PCP BH plan—an EMR template completed after each visit to document identification of BH concerns and recommendations for BH services.

### Sample and Treatment Group Definition

Children aged 4 to 18 years were included if they (1) were identified by their PCP as having a BH concern during the study period, (2) completed a baseline 17-item Pediatric Symptom Checklist (PSC-17) at the time of or after BH identification (index screening), and (3) completed at least 1 additional PSC-17 screener 6 to 18 months later. Children were included in a treatment group if they (1) received at least 1 of the 3 BH treatments within 90 days of BH identification (encounter with a BHC, encounter with a CHW, or prescription for psychotropic medication), and (2) completed their baseline PSC-17 before or on the date of BH treatment. Children were included in the control group if they qualified for the initial study sample but did not receive any of the 3 types of BH treatment during the study period (see the eFigure in Supplement 1 for the study sample flowchart).

### Measures

#### PSC-17

Behavioral symptoms were measured by the PSC-17, which is one of the most frequently used screeners for psychosocial problems in pediatrics.^23,24,25^ Seventeen items focus on multiple domains including attention, internalizing problems, and externalizing problems. Response options include never, sometimes, or often (scored 0, 1, and 2, respectively). Summing scores across items results in a total score (range of 0-34; score ≥15 indicates concern). The PSC-17 has displayed strong internal consistency and test-retest reliability and met the practical criteria for screening instruments.^23,26^ The 3 subscales—internalizing (5 items; score ≥5 indicates concern), externalizing (7 items; score ≥7 indicates concern), and attention (5 items; score ≥7 indicates concern)—capture distinct domains of psychosocial symptoms.^27^

#### Treatment Type

We evaluated 3 separate treatment types: (1) encounters with a BHC (identified through *Current Procedural Terminology* codes [eTable 1 in Supplement 1]), (2) encounters with a CHW, and (3) prescriptions for psychotropic medication. Each treatment group was compared with a nontreatment control group. Primary analyses compared any treatment to none; secondary analyses evaluated number of BHC and CHW encounters.

### Statistical Analysis

Because this study relied on evidence from the EMR, assignment to treatment and control groups was nonrandom. Therefore, patients who received treatment may differ from patients who did not receive treatment in important ways, such as having different baseline characteristics (eg, type and severity of disease and sociodemographic characteristics). To minimize potential selection bias, we used 1:2 nearest neighbor propensity score matching with replacement to match cases by sex, age, race and ethnicity, primary language, type of concern raised at visit, health-related social needs, having other types of BH treatment, and FQHC- and year-specific effects. Race and ethnicity categories included Hispanic, non-Hispanic Black, non-Hispanic White, and non-Hispanic other race or ethnicity (American Indian or Alaska Native, Asian, and race not specified). Pretreatment PSC-17 score was not included in the matching due to the potential for bias.^28,29^ Propensity score methods have been used to evaluate other evidence-based treatments,^30,31^ including measuring the impacts of integrated BH care.^20,32^

Our unit of analysis was the person-visit (eligible visit with BH screen score). The association of treatment with PSC-17 scores was estimated based on linear regression models with standard errors clustered at the patient level and analytic weights that accounted for the number of times an individual was selected into the propensity matched sample. The model structure was:

*Y_it_* = β_0_ + *β_1_Treatment_i_* + β_2_*Post_t_* + *β*_3_(*Treatment_i_* × *Post_t_*) + β_4_*Year_y_* + *X*′_it_β_5_ + μ_i_ + ε*_it_*

where *Y_it_* was the PSC-17 screen score of patient *i* at visit *t*. Independent variables included a binary indicator (β) for membership in the treatment group (*Treatment_i_*), a binary indicator for the posttreatment period (*Post_t_*, which equaled 0 for the pretreatment index screening and 1 for the posttreatment follow-up visits), and an interaction term between treatment status and post period (*Treatment_i_ × Post_t_*). The model also included fixed effects for year (*Year_y_*) and FQHC (μ_i_), a vector of patient-level covariates (*X’_it_*), and a residual error term (ε*_it_*) including all variables in the propensity score and time to follow-up screening to adjust for timing variability. Matching weights were applied in regression models to account for repeated selection of controls due to replacement.

Primary analyses defined treatment based on a minimum of 1 encounter or prescription. While this minimum may fall below therapeutic levels, it offered methodological advantages by avoiding additional confounders associated with whether families continued or desisted from treatment. As a secondary analysis, however, we reran our regression models to estimate the association of intensity of treatment encounters (ie, for a BHC encounter or CHW encounter) with PSC-17 scores by replacing the original interaction term with a new interaction term between categorical number of encounters and posttreatment period. Additionally, we examined the association of treatment with PSC-17 subscale scores (internalizing, externalizing, and attention) using the same regression framework. Data were analyzed in October 2024. We used Stata 18 (StataCorp) for all analyses, and a 2-sided *P* < .05 was considered significant.

## Results

Table 1 compares baseline characteristics among the 368 children with BHC encounters (215 female [58.4%]) with the 528 children in the control group (248 female [47.0%]) before and after propensity matching. Children with a BHC encounter were older (mean [SD] age, 11.7 [3.5] vs 10.9 [3.6] years), more likely to speak Spanish as their primary language (29.9% vs 19.5%), and more likely to have concerns identified by a practitioner, including depression (121 children [32.9%] vs 74 children [14.0%]) and anxiety (114 children [31.0%] vs 68 children [12.9%]). After matching, differences in observed baseline characteristics were minimized, with no statistically significant differences between the 2 groups (ie, *P* > .05). Comparisons were similar for CHW encounters and medication prescriptions (eTable 2 and eTable 3 in Supplement 1). For example, after matching, the analysis of BHC encounters compared a sample of children with a mean (SD) age of 11.8 (3.5) years and 206 children of female sex (58.7%) with a sample of children with a mean (SD) age of 11.7 (3.4) years and 204 children of female sex (56.7%). The final sample included 942 unique children, where 542 (57.5%) received any type of treatment and 400 (42.5%) were in the control group (eFigure in Supplement 1).

**Table 1.** Baseline Characteristics of Patients With a BHC Encounter (Treatment Group) vs Patients Without Any Treatment (Control Group) Before and After Propensity Score Matching

Characteristic	Patients, No. (%) (N= 942)^a^				
	Before propensity score matching		After propensity score matching		
	Children with a BHC encounter (n = 368)	Control group (n = 528)	Children with a BHC encounter (n = 351)	Control group (n = 359)	*P* value^b^
Site					
Site 1	76 (20.7)	181 (34.3)	74 (21.1)	73 (20.2)	.78
Site 2	95 (25.8)	161 (30.5)	89 (25.4)	109 (30.5)	.13
Site 3	137 (37.2)	107 (20.3)	131 (37.3)	119 (33.0)	.24
Site 4	60 (16.3)	79 (15.0)	57 (16.2)	58 (16.2)	>.99
Sex					
Female	215 (58.4)	248 (47.0)	206 (58.7)	204 (56.7)	.59
Male	153 (41.6)	280 (53.0)	145 (41.3)	155 (43.3)	.59
Age at visit, mean (SD), y	11.7 (3.5)	10.9 (3.6)	11.8 (3.5)	11.7 (3.4)	.77
Race and ethnicity					
Hispanic	161 (43.8)	161 (30.5)	154 (43.9)	148 (41.2)	.47
Non-Hispanic Black	74 (20.1)	158 (29.9)	70 (19.9)	92 (25.5)	.08
Non-Hispanic White	102 (27.7)	136 (25.8)	98 (27.9)	100 (27.9)	>.99
Non-Hispanic other^c^	31 (8.4)	73 (13.8)	29 (8.3)	19 (5.4)	.14
Primary language					
English	191 (51.9)	273 (51.7)	175 (49.9)	184 (51.3)	.71
Spanish	110 (29.9)	103 (19.5)	110 (31.3)	104 (29.1)	.51
Other	67 (18.2)	152 (28.8)	66 (18.8)	71 (19.7)	.77
Key health concerns					
Hyperactivity, inattention, or disruptive behavior	109 (29.6)	123 (23.3)	102 (29.1)	118 (32.8)	.29
Depression	121 (32.9)	74 (14.0)	151 (32.8)	120 (33.5)	.84
Anxiety	114 (31.0)	68 (12.9)	108 (30.8)	99 (27.6)	.36
Eating concerns	8 (2.2)	9 (1.7)	8 (2.3)	12 (3.4)	.37
Substance use or addiction risk	1 (0.3)	2 (0.4)	1 (0.3)	0	.32
Trauma or violence	7 (1.9)	3 (0.6)	7 (2.0)	10 (2.8)	.46
Family stress and/or stress reaction	27 (7.3)	23 (4.4)	24 (6.8)	28 (7.8)	.61
Emergency services^d^	57 (15.5)	45 (8.5)	54 (15.4)	51 (14.2)	.67
Chronic disease management (medical)	3 (0.8)	3 (0.6)	2 (0.6)	2 (0.6)	>.99
Social or material needs	10 (2.7)	34 (6.4)	8 (2.3)	6 (1.6)	.49
Other mental health concern	8 (2.2)	36 (6.8)	6 (1.7)	5 (1.4)	.76
Developmental concern	16 (4.3)	27 (5.1)	14 (4.0)	15 (4.3)	.85
Parent or caregiver mental health concern	66 (17.9)	97 (18.4)	62 (17.7)	60 (16.7)	.73
Early childhood concern (Building Resources and Access for Children’s Healthy Development)	0	1 (0.2)	0	0	NA
Safety or suicidal ideation concern	5 (1.4)	1 (0.2)	3 (0.9)	1 (0.3)	.32
School-related concern	9 (2.4)	6 (1.1)	8 (2.3)	10 (2.7)	.72
Health-related social needs					
Housing	9 (2.4)	21 (4.0)	9 (2.6)	13 (3.7)	.39
Food	29 (7.9)	62 (11.7)	26 (7.4)	25 (7.0)	.83
Transport	17 (4.6)	29 (5.5)	16 (4.6)	19 (5.3)	.66
Utilities	11 (3.0)	26 (4.9)	10 (2.8)	9 (2.4)	.72
Prior encounter with a community health worker	9 (2.4)	0	0	0	NA
Prior use of psychotropic medication	6 (1.6)	0	0	0	NA
Year					
2020	129 (35.1)	164 (31.1)	124 (35.3)	124 (34.5)	.81
2021	212 (57.6)	332 (62.9)	203 (57.8)	212 (59.1)	.73
2022	27 (7.3)	32 (6.1)	24 (6.8)	23 (6.4)	.82
2023	0	0	0	0	NA

Abbreviations: BHC, behavioral health clinician; NA, not applicable.

^a^ The total analytic sample consisted of 942 unique children, where the unique number of children with any type of treatment (either BHC encounter, community health worker encounter, or psychotropic medication use) was 542 and the unique number of children without any treatment was 400. For a detailed flowchart of the study sample, see the eFigure in Supplement 1.

^b^ *P* value was obtained in the 2-sided *t* test for equality of means in the 2 samples (treatment group vs control group).

^c^ American Indian or Alaska Native, Asian, and race not specified.

^d^ Section 12, Chapter 120 of the Massachusetts General Laws; early service practitioner, or Department of Children and Families filing.

Table 2 displays results of 3 sets of regression analyses measuring the association of receiving each integrated treatment with PSC-17 screening scores. Negative estimates in PSC-17 scores reflect reductions in symptoms and therefore improvement in functioning. In each set of regression analyses, a primary analysis evaluated differences between children who did and did not receive treatment, holding other covariates constant. Compared with children who received no treatment, baseline PSC-17 scores were, on average, 2.06 points (95% CI, 1.03 to 3.09 points) higher among children who had at least 1 BHC encounter, 1.46 points (95% CI, 0.29 to 2.63 points) higher among children who had at least 1 CHW encounter, and 4.06 points (95% CI, 2.76 to 5.36 points) higher among children who received a prescription for medication, indicating that children with poorer psychosocial symptoms at baseline tended to receive treatment. Over time, there was no significant change in PSC-17 scores experienced by children in the control group. However, receipt of treatment was associated with improvement in symptoms. Receiving at least 1 encounter with a BHC was associated with a 1.51 (95% CI, −2.65 to −0.37)–point reduction in PSC-17 scores among children in the treatment group, compared with children in the control group. We observed no statistical association of receipt of at least 1 CHW encounter with PSC-17 score (−0.53 points; 95% CI, −1.86 to 0.80 points). Finally, receipt of a prescription for psychotropic medication was associated with a reduction in PSC-17 scores among children in treatment group (−2.21 points; 95% CI, −3.89 to −0.54 points), compared with children in the control group. Descriptive analyses showed no systematic differences in follow-up duration by treatment status (eTable 4 in Supplement 1), suggesting low risk of confounding.

**Table 2.** Association of Receiving Treatment (BHC Encounter, CHW Encounter, or Medication Use) With PSC-17 Screening Scores Among Children in TEAM UP Federally Qualified Health Centers

Analysis type	PSC-17, coefficient (95% CI)^a^		
	BHC encounter	CHW encounter	Medication use
**Primary regression analyses with an interaction term between treatment status and postteatment status** ^b^			
Treatment group			
Control	0 [Reference]	0 [Reference]	0 [Reference]
Treatment	2.06 (1.03 to 3.09)	1.46 (0.29 to 2.63)	4.06 (2.76 to 5.36)
Treatment period			
Pretreatment	0 [Reference]	0 [Reference]	0 [Reference]
Posttreatment	−0.64 (−2.59 to 1.31)	−0.64 (−2.93 to 1.65)	−1.51 (−4.15 to 1.12)
Treatment group × treatment period			
Control group × posttreatment	0 [Reference]	0 [Reference]	0 [Reference]
Treatment group × posttreatment	−1.51 (−2.65 to −0.37)	−0.53 (−1.86 to 0.80)	−2.21 (−3.89 to −0.54)
*R* ^2^	0.15	0.25	0.27
No. of observations	2283	1399	768
**Secondary regression analyses with interaction terms between for the number of encounters and posttreatment status** ^b^			
No. of encounters			
0	0 [Reference]	0 [Reference]	NA
1	1.89 (0.63 to 3.13)	1.48 (0.08 to 2.87)	NA
2	1.21 (−0.58 to 3.00)	0.66 (−1.38 to 2.71)	NA
≥3	2.38 (1.14 to 3.61)	1.87 (0.24 to 3.51)	NA
Treatment period			
Pretreatment	0 [Reference]	0 [Reference]	NA
Posttreatment	−0.64 (−2.62 to 1.33)	−0.40 (−2.73 to 1.93)	NA
No. of encounters × treatment period			
0 encounters × posttreatment	0 [Reference]	0 [Reference]	NA
1 Encounter × posttreatment	−1.03 (−2.49 to 0.44)	−0.63 (−2.22 to 0.96)	NA
2 Encounters × posttreatment	−2.17 (−4.03 to −0.31)	0.87 (−1.22 to 2.96)	NA
≥3 Encounters × posttreatment	−1.70 (−3.03 to −0.37)	−1.16 (−2.98 to 0.66)	NA
*R* ^2^	0.15	0.25	NA
No. of observation	2283	1399	NA

Abbreviations: BHC, behavioral health clinician; CHW, community health worker; NA, not applicable; PSC-17, Pediatric Symptom Checklist-17; TEAM UP, Transforming and Expanding Access to Mental Health Universally in Pediatrics.

^a^ A negative coefficient of the interaction term means that the PSC-17 score was lower (ie, psychosocial symptom was improved) for patients with treatment in the posttreatment period compared with patients without any treatment.

^b^ The model included year-specific effects, federally qualified health centers fixed effects, and a vector of patient-level covariates such as sex, age, race and ethnicity, primary language, type of concern raised at visit (eg, hyperactivity, inattention, or disruptive behavior; depression; anxiety; eating concerns; substance use or additional risk; trauma or violence; family stress and/or stress reaction; emergency services; chronic disease management; social or material needs; other behavioral health concern; developmental concern; parent or caregiver mental health concern; early childhood concern; safety or suicidal ideation concern; and school-related concern), health-related social needs (eg, needs for housing, food, transportation, and utilities), having other types of behavioral health treatment, and time to follow-up screening after the index screening date.

For BHC and CHW encounters, secondary regression analyses examined association of the number of encounters with BH symptom scores holding other covariates constant (Table 2). While PSC-17 scores were not associated with receiving just 1 BHC encounter, improvements in PSC-17 scores were significantly associated with receiving 2 BHC encounters (−2.17 points; 95% CI, −4.03 to −0.31 points) or 3 or more BHC encounters (−1.70 points; 95% CI, −3.03 to −0.37 points). There was no statistically significant association of CHW encounters with BH symptom scores at any level of treatment intensity.

Additional analyses examined specific PSC-17 domains. Receiving at least 1 encounter with a BHC was associated with a 0.77-point reduction (95% CI, −1.26 to −0.28 points) in externalizing scores compared with the control group (eTable 5 in Supplement 1). Receipt of medication was associated with a 0.92-point reduction (95% CI, −1.72 to −0.13 points) in externalizing scores and a 0.83-point reduction (95% CI, −1.63 to −0.03 points) in attention scores. Descriptive analyses suggest that symptom improvements generally aligned with the type of presenting concern and treatment received (eTable 6 in Supplement 1).

We conducted additional analyses to characterize BH concerns as identified by PCPs. Children identified with BH concerns had higher PSC-17 scores, yet mean scores fell below clinical cutoffs (eTable 7 in Supplement 1),^33^ thus limiting room for improvement and likely attenuating estimates of association. Further analyses supported PCP identification as a marker of psychosocial risk. Among children identified with BH concerns, PSC-17 subscale elevations aligned with specific concerns and diagnoses (eg, attention scores for hyperactivity, inattention, or disruptive behavior, and internalizing scores for anxiety or depression) (eTable 8 in Supplement 1). Even among children with nonelevated total PSC-17 scores, many had subscale elevations aligned with concerns (eTable 9 in Supplement 1). Lastly, given the small number of CHW encounters, likely due to underdocumentation or a reporting issue, we conducted a sensitivity analysis excluding data from site 3, which showed results consistent with the main analysis, supporting the robustness of our findings (eTable 10 in Supplement 1).

## Discussion

This observational cohort study provides evidence for the association of integrated pediatric BH care at FQHCs using the TEAM UP model with BH outcomes. Encounters with BHCs and psychotropic medication prescriptions were significantly associated with improved BH symptoms among children with identified BH concerns; however, there was no statistical association with CHW encounters. Findings suggest a dose effect for BHC encounters, where having 2 or more visits with a BHC was underlying the observed positive improvements. To our knowledge, this is the first study to use a nonrandomized trial to examine the association of integrated pediatric BH services with BH symptoms.

The magnitude of PSC-17 score reductions suggest that, on average, 1 or 2 symptoms improved, with greater declines among children treated with medication, who often presented with more severe concerns. Alignment between symptom improvements, the type of presenting concern, and treatment received (eTable 6 in Supplement 1) support the clinical meaningfulness of these findings. Several hypotheses may explain results. Children may have benefited directly from psychotherapy provided by BHCs or indirectly through coordination of care with CHWs and PCPs. Being able to receive prescriptions and medication management within FQHCs may have helped children who would otherwise be referred elsewhere and would have difficulty finding appropriate practitioners. In contrast, failure to find associations of CHW encounters with changes in child symptoms motivates additional research. For example, CHWs’ focus on navigation and addressing health-related social needs suggest that this service might best be evaluated with respect to other, more targeted outcomes that may better explain its strong support among PCPs and BHCs.^34^

This study offers 3 key contributions. First, findings suggest that prior RCTs on the effectiveness of psychotherapy and psychotropic medications^7,11,12,35,36,37,38,39^ generalize to TEAM UP. Evidence that psychotropic medications are associated with improved psychosocial outcomes in a diverse patient population in empirical settings is relatively rare^12,40,41,42,43^ despite the high and increasing prescription rates for psychotropic medications for children,^35,44,45^ and most meta-analyses and systemic reviews of psychotropic medications focus on specific diagnoses, such as attention-deficit/hyperactivity disorder, depression, and anxiety.^46,47,48,49,50,51^ In addition, prior studies of outpatient BH treatment^30,31,52,53,54,55^ typically included samples from general community-based outpatient clinics. In contrast, our study included children from FQHCs who were diverse with respect to presenting concern and demographic characteristics.

Second, findings extend the evidence based on the TEAM UP intervention. Prior analyses based on claims data offer evidence that TEAM UP is associated with increased provision of BH services.^20,21^ However, claims data alone offer limited insights into health outcomes. Current findings document associations of TEAM UP BH services with improved psychosocial outcomes, thus providing additional evidence regarding an important link in how TEAM UP may influence population health (Figure).

Third, our study focuses on specific elements of BH care. Like other such models, TEAM UP is a complex intervention with many moving parts. Improving the model over time requires decisions about whether specific elements should be continued, modified, or deimplemented. In this light, evidence for the association with BHC care and medication management support decisions to continue focusing resources on these interventions. In addition, the dose effect, which is consistent with a recent systematic review that documented improvements after 4 visits,^56^ suggests that decisions promoting sustained engagement with BHCs beyond a single visit could enhance outcomes. Together, these contributions add to the evidence base on models of care that may improve pediatric BH.

### Limitations

We note several limitations. First, EMRs did not include information on health care utilization that occurred outside FQHCs, including affiliated specialty clinics and school services. Second, there were limited preperiod data to test the preintervention parallel trend assumption of a difference-in-differences approach; thus, our approach is best described as a pre-post design with propensity matched treatment and control groups. To minimize confounding effects of selection bias, we employed matching techniques to ensure baseline balance between the treatment and control groups; however, estimates should not be interpreted causally. Third, even though PSC-17 scores have high serial correlation within a child, results may be subject to bias due to regression to the mean.^57^ Fourth, variation in the timing of PSC-17 follow-up assessments (6 to 18 months) may have introduced heterogeneity in outcome measurement. However, we adjusted for days between assessments in all models, and descriptive analyses showed no systemic differences in follow-up duration by treatment status, suggesting low risk of confounding. Moreover, any variation likely biases estimates toward the null because associations may attenuate over time. Fifth, because standards for a minimal clinically important difference have not been established for the PSC-17, clinical significance is difficult to estimate. However, we note that the PSC-17 is designed as a screening tool—not to be sensitive to changes in symptoms over time. Therefore, even robust clinical improvement may result in small statistical differences.^58^ Prior literature reported change over time for the 35-item PSC among children with elevated scores, which resulted in larger changes in scores over time,^24,26,53^ with 1 longitudinal study in outpatient pediatric clinics documenting an approximate 3.2-point reduction in PSC-35 scores among children referred for BH care.^59^ In contrast, we reported changes over time but in comparison with a control group and used the PSC-17 rather than the 35-item PSC. Sixth, we report results for all children identified with BH concerns—not just those with elevated screening scores. Children identified by their PCPs as having BH concerns had higher PSC-17 scores, yet means fell below clinical cutoffs,^33^ thus limiting room for improvement and likely attenuating estimates of association.

## Conclusions

In this cohort study of children at FQHCs implementing BH integration, we found receiving services from a BHC or receiving psychotropic medications in the context of integrated care were associated with improved BH symptoms. These findings suggest that BH integration within FQHCs may be a promising vehicle for expanding the reach of pediatric BH care and for improving the health outcomes within marginalized populations.

## Notes

Supplement 1. **eFigure.** Flowchart for Study Sample (Unique Number of Children) **eTable 1.** List of Current Procedural Terminology (CPT) Codes Used to Define Encounters With a BHC **eTable 2.** Baseline Characteristics of Patients With a CHW Encounter (Treatment Group) vs. Patients Without Any Treatment (Control Group) Before and After Propensity Score Matching **eTable 3.** Baseline Characteristics of Patients With a Psychotropic Medication Use (Treatment Group) vs. Patients Without Any Treatment (Control Group) Before and After Propensity Score Matching **eTable 4.** Distribution of Days Between PSC-17 Assessments by Treatment Status and Intensity **eTable 5.** Association Between Receiving Treatment and the PSC-17 Screen Sub-Scores (Internalizing, Externalizing, and Attention Scores) Among Children in TEAM UP FQHCs **eTable 6.** Changes in PSC-17 Item Scores From Baseline to Follow-Up by Selected Treatment and Diagnosis Type **eTable 7.** Distribution of PSC-17 Scores by Behavioral Health (BH) Concern Identification Status **eTable 8.** Distribution of PSC-17 Scores by Type of Behavioral Health (BH) Concern/Diagnosis Among Children Identified by Their Primary Care Provider as Having a BH Concern **eTable 9.** Documented Behavioral Health Concerns/Diagnoses Among Children With Non-Elevated PSC-17 Scores Who Were Identified by Their PCP as Having a BH Concern **eTable 10.** Association Between CHW Encounter and the PSC-17 Screen Scores Among Children in TEAM UP FQHCs (a Sensitivity Analysis Excluding Data From Site 3)

Supplement 2.
Data Sharing Statement
