# Supplementary material for: Integrated Behavioral Health Services and Psychosocial Symptoms in Children
Source: JAMA Netw Open. 2025 Sep 16;8(9):e2532020. doi: 10.1001/jamanetworkopen.2025.32020 (PMC12441876; doi:10.1001/jamanetworkopen.2025.32020)
Supplement: Supplement 1. — eFigure. Flowchart for Study Sample (Unique Number of Children) eTable 1. List of Current Procedural Terminology (CPT) Codes Used to Define Encounters With a BHC eTable 2. Baseline Characteristics of Patients With a CHW Encounter (Treatment Group) vs. Patients Without Any Treatment (Control Group) Before and After Propensity Score Matching eTable 3. Baseline Characteristics of Patients With a Psychotropic Medication Use (Treatment Group) vs. Patients Without Any Treatment (Control Group) Before and After Propensity Score Matching eTable 4. Distribution of Days Between PSC-17 Assessments by Treatment Status and Intensity eTable 5. Association Between Receiving Treatment and the PSC-17 Screen Sub-Scores (Internalizing, Externalizing, and Attention Scores) Among Children in TEAM UP FQHCs eTable 6. Changes in PSC-17 Item Scores From Baseline to Follow-Up by Selected Treatment and Diagnosis Type eTable 7. Distribution of PSC-17 Scores by Behavioral Health (BH) Concern Identification Status eTable 8. Distribution of PSC-17 Scores by Type of Behavioral Health (BH) Concern/Diagnosis Among Children Identified by Their Primary Care Provider as Having a BH Concern eTable 9. Documented Behavioral Health Concerns/Diagnoses Among Children With Non-Elevated PSC-17 Scores Who Were Identified by Their PCP as Having a BH Concern eTable 10. Association Between CHW Encounter and the PSC-17 Screen Scores Among Children in TEAM UP FQHCs (a Sensitivity Analysis Excluding Data From Site 3) [file jamanetwopen-e2532020-s001.pdf]

Supplemental Online Content

Kim J, Cole MB, Rosenberg J, Morris A, Feinberg E, Sheldrick RC. Integrated behavioral health services and psychosocial symptoms in children. *JAMA Netw. Open*. 2025;8(9):e2532020. doi:10.1001/jamanetworkopen.2025.32020

eFigure. Flowchart for Study Sample (Unique Number of Children)

eTable 1. List of Current Procedural Terminology (CPT) Codes Used to Define Encounters With a BHC

eTable 2. Baseline Characteristics of Patients With a CHW Encounter (Treatment Group) vs. Patients Without Any Treatment (Control Group) Before and After Propensity Score Matching

eTable 3. Baseline Characteristics of Patients With a Psychotropic Medication Use (Treatment Group) vs. Patients Without Any Treatment (Control Group) Before and After Propensity Score Matching

eTable 4. Distribution of Days Between PSC-17 Assessments by Treatment Status and Intensity

eTable 5. Association Between Receiving Treatment and the PSC-17 Screen Sub-Scores (Internalizing, Externalizing, and Attention Scores) Among Children in TEAM UP FQHCs

eTable 6. Changes in PSC-17 Item Scores From Baseline to Follow-Up by Selected Treatment and Diagnosis Type

eTable 7. Distribution of PSC-17 Scores by Behavioral Health (BH) Concern Identification Status

eTable 8. Distribution of PSC-17 Scores by Type of Behavioral Health (BH) Concern/Diagnosis Among Children Identified by Their Primary Care Provider as Having a BH Concern

eTable 9. Documented Behavioral Health Concerns/Diagnoses Among Children With Non-Elevated PSC-17 Scores Who Were Identified by Their PCP as Having a BH Concern

eTable 10. Association Between CHW Encounter and the PSC-17 Screen Scores Among Children in TEAM UP FQHCs (a Sensitivity Analysis Excluding Data From Site 3)

This supplemental material has been provided by the authors to give readers additional information about their work.

© 2025 Kim J et al. *JAMA Network Open*.

Supplemental Figure 1. Flowchart for study sample (unique number of children)

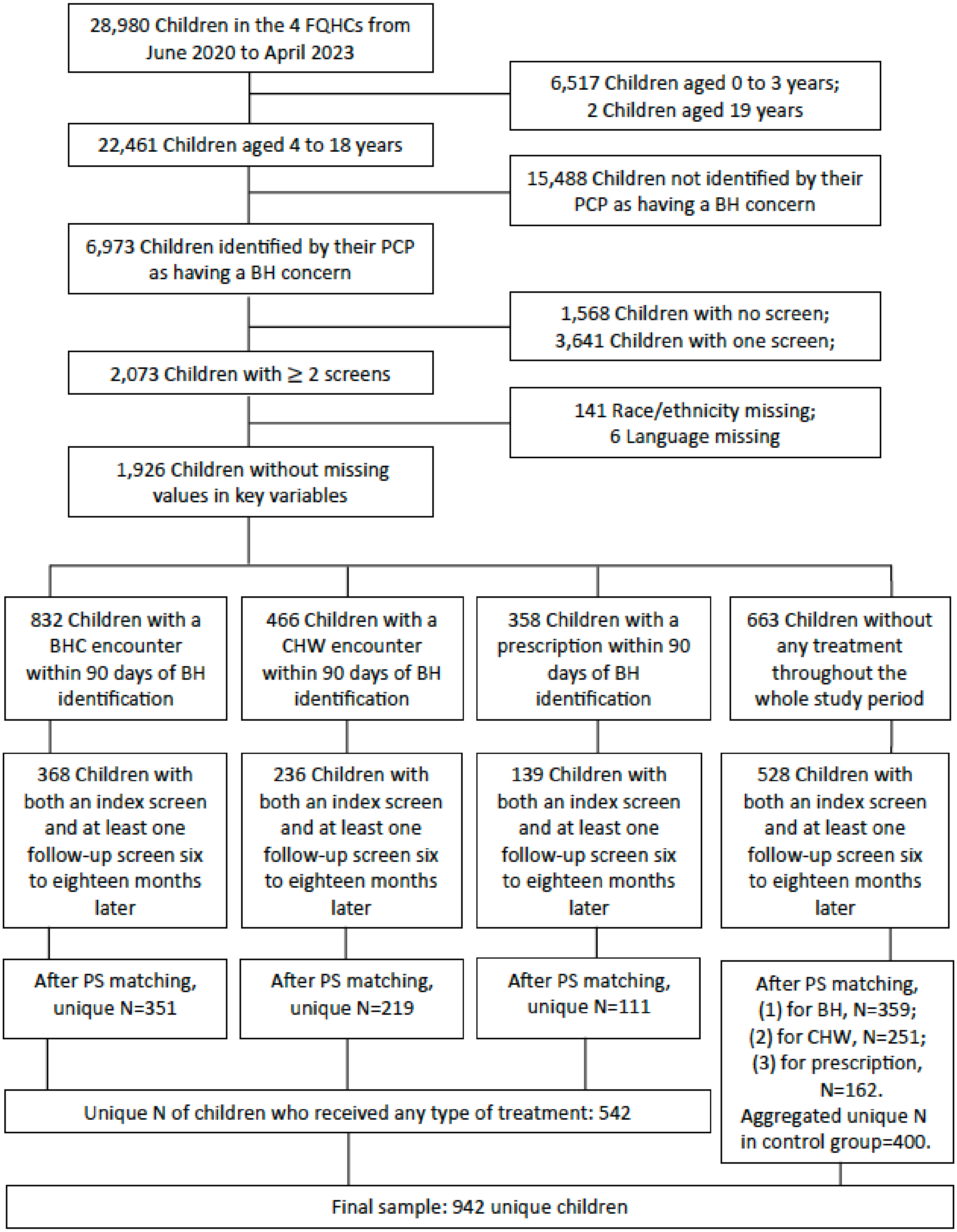

© 2025 Kim J et al. *JAMA Network Open*.

eTable 1. List of Current Procedural Terminology (CPT) codes used to define encounters with a BHC

Treatment type	CPT codes
Encounters with a BHC	**Psychotherapy**: 90832, 90833, 90834, 90835, 90836, 90837, 90838, 90846, 90847, 90849, 90804, 90805, 90806, 90807, 90808, 90816, 90818, 90821, 96150, 96151, 96152, 96153, 96154, 96155, H2019, H2020, T1040;<br>**BH intake**: 90791, 90792, 90801, 90802;<br>**Other BH services**: 90882, 90887, 90839, 90840, H2011, 90853, 99211, 99212, 99213, 99214, 99215, 99201, 99202, 99203, 99204, 99205, H0046, H0032, 96111.

© 2025 Kim J et al. *JAMA Network Open*.

eTable 2. Baseline characteristics of patients with a CHW encounter (treatment group) vs. patients without any treatment (control group) before and after propensity score matching

	Before matching		After matching		
	Treatment group (n=236)	Control group (n=528)	Treatment group (n=219)	Control group (n=251)	P-value [a]
Site, No. (%)					
Site 1	49 (20.8)	181 (34.3)	47 (21.5)	50 (20.1)	0.725
Site 2	107 (45.3)	161 (30.5)	98 (44.7)	115 (45.7)	0.848
Site 3	11 (4.7)	107 (20.3)	7 (3.2)	12 (4.8)	0.394
Site 4	69 (29.2)	79 (15.0)	67 (30.6)	74 (29.5)	0.795
Gender, No. (%)					
Female	123 (52.1)	248 (47.0)	114 (52.1)	120 (47.9)	0.391
Male	113 (47.9)	280 (53.0)	105 (47.9)	131 (52.1)	0.391
Age at visit (in year), mean (SD)	10.6 (3.6)	10.9 (3.6)	10.5 (3.6)	10.9 (3.6)	0.287
Race/ethnicity, No. (%)					
Non-Hispanic white	56 (23.7)	136 (25.8)	53 (24.2)	58 (23.1)	0.779
Non-Hispanic black	58 (24.6)	158 (29.9)	53 (24.2)	55 (21.9)	0.572
Non-Hispanic other	34 (14.4)	73 (13.8)	32 (14.6)	39 (15.5)	0.790
Hispanic	88 (37.3)	161 (30.5)	81 (37.0)	99 (39.5)	0.590
Language, No. (%)					
English	135 (57.2)	273 (51.7)	125 (57.1)	141 (56.2)	0.848
Spanish	51 (21.6)	103 (19.5)	46 (21.0)	49 (19.4)	0.678
Other	50 (21.2)	152 (28.8)	48 (21.9)	61 (24.4)	0.534
Key issues, No. (%)					
Hyperactivity, inattention, or disruptive behavior	81 (34.3)	123 (23.3)	74 (33.8)	77 (30.8)	0.508
Depression	41 (17.4)	74 (14.0)	34 (15.5)	51 (20.3)	0.192
Anxiety	49 (20.8)	68 (12.9)	44 (20.1)	49 (19.6)	0.905
Eating issues	2 (0.8)	9 (1.7)	1 (0.5)	1 (0.5)	1.000
Substance use/addiction risk	0 (0.0)	2 (0.4)	0 (0.0)	0 (0.0)	.
Trauma/violence	2 (0.8)	3 (0.6)	2 (0.9)	3 (1.1)	0.813
Family stress and/or stress reaction	11 (4.7)	23 (4.4)	9 (4.1)	17 (6.6)	0.244
Emergency services (section 12, ESP, DCF filing, etc.)	38 (16.1)	45 (8.5)	35 (16.0)	36 (14.4)	0.642
Chronic disease management (medical)	2 (0.8)	3 (0.6)	1 (0.5)	2 (0.7)	0.752
Social/material needs	17 (7.2)	34 (6.4)	15 (6.8)	21 (8.2)	0.588
Other mental health concern	22 (9.3)	36 (6.8)	19 (8.7)	26 (10.3)	0.569
Developmental concern	6 (2.5)	27 (5.1)	5 (2.3)	5 (1.8)	0.737
Parent/caregiver mental health concern	72 (30.5)	97 (18.4)	70 (32.0)	84 (33.6)	0.722
Early childhood concern (BRANCH)	0 (0.0)	1 (0.2)	0 (0.0)	0 (0.0)	.
Safety/suicidal ideation concern	1 (0.4)	1 (0.2)	0 (0.0)	0 (0.0)	.

© 2025 Kim J et al. *JAMA Network Open*.

School related concern	7 (3.0)	6 (1.1)	5 (2.3)	7 (2.7)	0.761
Health-related social needs, No. (%)					
Housing	11 (4.7)	21 (4.0)	11 (5.0)	14 (5.5)	0.831
Food	45 (19.1)	62 (11.7)	41 (18.7)	66 (26.3)	0.059
Transport	11 (4.7)	29 (5.5)	10 (4.6)	5 (2.1)	0.143
Utilities	12 (5.1)	26 (4.9)	12 (5.5)	17 (6.6)	0.617
Prior encounter with a BHC, No. (%)	15 (5.1)	0 (0.0)	0 (0.0)	0 (0.0)	.
Prior use of psychotropic medication, No. (%)	4 (1.7)	0 (0.0)	0 (0.0)	0 (0.0)	.
Year, No. (%)					
2020	90 (38.1)	164 (31.1)	85 (38.8)	100 (39.7)	0.845
2021	133 (56.4)	332 (62.9)	123 (56.2)	140 (55.9)	0.962
2022	13 (5.5)	32 (6.1)	11 (5.0)	11 (4.3)	0.735
2023	0 (0.0)	0 (0.0)	0 (0.0)	0 (0.0)	.

[a] P-value was obtained in the t-test for equality of means in the two samples (treatment group vs. control group).

© 2025 Kim J et al. *JAMA Network Open*.

eTable 3. Baseline characteristics of patients with a psychotropic medication use (treatment group) vs. patients without any treatment (control group) before and after propensity score matching

	Before matching		After matching		
	Treatment group (n=139)	Control group (n=528)	Treatmen t group (n=111)	Control group (n=162)	P-value [a]
Site, No. (%)					
Site 1	27 (19.4)	181 (34.3)	23 (20.7)	31 (19.4)	0.803
Site 2	22 (15.8)	161 (30.5)	21 (18.9)	37 (22.5)	0.510
Site 3	42 (30.2)	107 (20.3)	30 (27.0)	43 (26.6)	0.940
Site 4	48 (34.5)	79 (15.0)	37 (33.3)	51 (31.5)	0.776
Gender, No. (%)					
Female	62 (44.6)	248 (47.0)	47 (42.3)	64 (39.6)	0.684
Male	77 (55.4)	280 (53.0)	64 (57.7)	98 (60.4)	0.684
Age at visit (in year), mean (SD)	11.7 (3.2)	10.9 (3.6)	11.5 (3.2)	11.5 (3.7)	0.947
Race/ethnicity, No. (%)					
Non-Hispanic white	60 (43.2)	136 (25.8)	45 (40.5)	56 (34.7)	0.370
Non-Hispanic black	23 (16.5)	158 (29.9)	20 (18.0)	39 (23.9)	0.286
Non-Hispanic other	6 (4.3)	73 (13.8)	6 (5.4)	10 (6.3)	0.776
Hispanic	50 (36.0)	161 (30.5)	40 (36.0)	57 (35.1)	0.889
Language, No. (%)					
English	91 (65.5)	273 (51.7)	72 (64.9)	110 (68.0)	0.621
Spanish	31 (22.3)	103 (19.5)	24 (21.6)	31 (19.4)	0.679
Other	17 (12.2)	152 (28.8)	15 (13.5)	21 (12.6)	0.843
Key issues, No. (%)					
Hyperactivity, inattention, or disruptive behavior	79 (56.8)	123 (23.3)	65 (58.6)	91 (56.3)	0.736
Depression	38 (27.3)	74 (14.0)	25 (22.5)	42 (25.7)	0.585
Anxiety	29 (20.9)	68 (12.9)	24 (21.6)	38 (23.4)	0.749
Eating issues	3 (2.2)	9 (1.7)	1 (0.9)	3 (1.8)	0.563
Substance use/addiction risk	0 (0.0)	2 (0.4)	0 (0.0)	0 (0.0)	.
Trauma/violence	3 (2.2)	3 (0.6)	3 (2.7)	4 (2.7)	1.000
Family stress and/or stress reaction	8 (5.8)	23 (4.4)	3 (2.7)	4 (2.3)	0.830
Emergency services (section 12, ESP, DCF filing, etc.)	21 (15.1)	45 (8.5)	17 (15.3)	16 (9.9)	0.227
Chronic disease management (medical)	2 (1.4)	3 (0.6)	1 (0.9)	1 (0.5)	0.684
Social/material needs	2 (1.4)	34 (6.4)	1 (0.9)	1 (0.5)	0.684
Other mental health concern	3 (2.2)	36 (6.8)	3 (2.7)	3 (1.8)	0.653
Developmental concern	3 (2.2)	27 (5.1)	3 (2.7)	2 (1.4)	0.477
Parent/caregiver mental health concern	52 (37.4)	97 (18.4)	39 (35.1)	48 (29.7)	0.392
Early childhood concern (BRANCH)	0 (0.0)	1 (0.2)	0 (0.0)	0 (0.0)	.
Safety/suicidal ideation concern	3 (2.2)	1 (0.2)	0 (0.0)	0 (0.0)	.

© 2025 Kim J et al. *JAMA Network Open*.

School related concern	1 (0.7)	6 (1.1)	1 (0.9)	0 (0.0)	0.318
Health-related social needs, No. (%)					
Housing	5 (3.6)	21 (4.0)	4 (3.6)	4 (2.7)	0.702
Food	9 (6.5)	62 (11.7)	9 (8.1)	17 (10.4)	0.564
Transport	6 (4.3)	29 (5.5)	5 (4.5)	7 (4.5)	1.000
Utilities	1 (0.7)	26 (4.9)	1 (0.9)	1 (0.9)	1.000
Prior encounter with a BHC, No. (%)	15 (10.8)	0 (0.0)	0 (0.0)	0 (0.0)	.
Prior encounter with a CHW, No. (%)	1 (0.7)	0 (0.0)	0 (0.0)	0 (0.0)	.
Year, No. (%)					
2020	75 (54.0)	164 (31.1)	58 (52.3)	101 (62.2)	0.137
2021	59 (42.4)	332 (62.9)	49 (44.1)	58 (35.6)	0.195
2022	5 (3.6)	32 (6.1)	4 (3.6)	3 (2.3)	0.552
2023	0 (0.0)	0 (0.0)	0 (0.0)	0 (0.0)	.

[a] P-value was obtained in the t-test for equality of means in the two samples (treatment group vs. control group).

© 2025 Kim J et al. *JAMA Network Open*.

eTable 4. Distribution of days between PSC-17 assessments by treatment status and intensity

Follow-up duration	Control	BHC encounter				Medication			
		All	1	2	3+	All	1	2	3+
181-211 days	3	32	5	3	24	7	0	1	6
212-244 days	2	23	4	0	19	3	0	0	3
245-275 days	2	26	5	2	19	5	0	1	4
276-306 days	4	25	6	2	17	8	1	0	7
307-337 days	3	24	2	4	17	7	1	0	6
338-368 days	65	38	16	4	17	12	6	1	4
369-399 days	211	137	50	21	59	60	21	11	16
400-430 days	91	76	26	11	35	32	6	5	16
431-461 days	56	54	15	6	32	26	6	2	14
462-492 days	44	33	8	6	17	10	2	1	6
493-524 days	30	26	6	0	19	11	4	2	3
525-545 days	20	12	3	0	9	4	2	0	2

Note: Median follow-up duration is 195 among those in control group, 261 among those with a BHC encounter, and 307 among those with medication use.

© 2025 Kim J et al. *JAMA Network Open*.

eTable 5. Association between receiving treatment and the PSC-17 screen sub-scores (internalizing, externalizing, and attention scores) among children in TEAM UP FQHCs

	Coefficient[a] (95% CI[b])		
	Treatment type: BHC encounter	Treatment type: CHW encounter	Treatment type: Medication use
Panel A. Primary regression analyses with an interaction term between treatment status & post status [c]			
Outcome: Internalizing score			
Treatment			
Control group	(Reference)	(Reference)	(Reference)
Treated group	0.77 (0.34, 1.20)	0.29 (-0.14, 0.72)	1.16 (0.70, 1.62)
Post			
Pre	(Reference)	(Reference)	(Reference)
Post	-0.62 (-1.51, 0.28)	-0.70 (-1.61, 0.22)	-0.29 (-1.51, 0.94)
Treatment×Post			
Control group×Post	(Reference)	(Reference)	(Reference)
Treated group×Post	-0.36 (-0.92, 0.20)	-0.03 (-0.61, 0.54)	-0.51 (-1.16, 0.15)
Outcome: Externalizing score			
Treatment			
Control group	(Reference)	(Reference)	(Reference)
Treated group	0.84 (0.42, 1.26)	0.55 (0.01, 1.09)	1.05 (0.38, 1.72)
Post			
Pre	(Reference)	(Reference)	(Reference)
Post	-0.02 (-0.79, 0.75)	-0.35 (-1.42, 0.71)	-0.23 (-1.19, 0.72)
Treatment×Post			
Control group×Post	(Reference)	(Reference)	(Reference)
Treated group×Post	-0.77 (-1.26, -0.28)	-0.38 (-1.01, 0.26)	-0.92 (-1.72, -0.13)
Outcome: Attention score			
Treatment			
Control group	(Reference)	(Reference)	(Reference)
Treated group	0.37 (-0.11, 0.85)	0.55 (0.03, 1.07)	1.68 (1.03, 2.33)
Post			
Pre	(Reference)	(Reference)	(Reference)
Post	-0.04 (-1.04, 0.96)	0.43 (-0.73, 1.59)	-0.83 (-1.63, -0.03)
Treatment×Post			
Control group×Post	(Reference)	(Reference)	(Reference)
Treated group×Post	-0.31 (-0.82, 0.20)	-0.17 (-0.75, 0.42)	-0.83 (-1.63, -0.03)
Panel B. Secondary regression analyses with interaction terms between for the number of encounters & post status [c]			
Outcome: Internalizing score			
# Encounters			
0 encounter	(Reference)	(Reference)	

© 2025 Kim J et al. *JAMA Network Open*.

1 encounter	0.54 (0.04, 1.05)	0.29 (-0.21, 0.79)	
2 encounters	0.65 (-0.17, 1.46)	0.48 (-0.22, 1.18)	
≥3 encounters	0.94 (0.44, 1.44)	0.19 (-0.42, 0.81)	
Post status			
Pre	(Reference)	(Reference)	
Post	-0.57 (-1.47, 0.34)	-0.73 (-1.65, 0.18)	
# Encounters×Post			
0 encounter×Post	(Reference)	(Reference)	
1encounter×Post	-0.25 (-0.91, 0.40)	-0.10 (-0.75, 0.56)	
2 encounters×Post	-0.29 (-1.16, 0.59)	0.11 (-0.77, 0.99)	
≥3 encounters×Post	-0.45 (-1.09, 0.19)	-0.06 (-0.84, 0.72)	
Outcome: Externalizing score			
# Encounters			
0 encounter	(Reference)	(Reference)	
1 encounter	0.93 (0.36, 1.51)	0.59 (-0.05, 1.23)	
2 encounters	0.54 (-0.28, 1.35)	-0.20 (-1.13, 0.74)	
≥3 encounters	0.86 (0.35, 1.36)	0.89 (0.11, 1.67)	
Post status			
Pre	(Reference)	(Reference)	
Post	-0.05 (-0.82, 0.73)	-0.16 (-1.25, 0.94)	
# Encounters×Post			
0 encounter×Post	(Reference)	(Reference)	
1encounter×Post	-0.45 (-1.14, 0.23)	-0.53 (-1.27, 0.21)	
2 encounters×Post	-0.82 (-1.65, 0.00)	0.56 (-0.29, 1.41)	
≥3 encounters×Post	-0.94 (-1.49, -0.39)	-0.55 (-1.48, 0.37)	
Outcome: Attention score			
# Encounters			
0 encounter	(Reference)	(Reference)	
1 encounter	0.34 (-0.25, 0.92)	0.53 (-0.08, 1.14)	
2 encounters	-0.05 (-0.91, 0.82)	0.42 (-0.50, 1.37)	
≥3 encounters	0.48 (-0.09, 1.05)	0.63 (-0.12, 1.38)	
Post status			
Pre	(Reference)	(Reference)	
Post	-0.07 (-1.08, 0.94)	0.46 (-0.76, 1.67)	
# Encounters×Post			
0 encounter×Post	(Reference)	(Reference)	
1encounter×Post	-0.27 (-0.90, 0.36)	-0.01 (-0.71, 0.69)	
2 encounters×Post	-0.93 (-1.82, -0.04)	0.00 (-0.95, 0.94)	
≥3 encounters×Post	-0.24 (-0.84, 0.37)	-0.61 (-1.38, 0.17)	
Number of Observation	2,283	1,399	768

© 2025 Kim J et al. *JAMA Network Open*.

[a] A negative coefficient of the interaction term means that the PSC score was lower (psychosocial functioning was improved) for patients with treatment after the treatment compared with patients without any treatment.
[b] 95% confidence interval (CI) in brackets.
[c] The model included year-specific effects, FQHC fixed effects, and a vector of patient-level covariates such as sex, age, race/ethnicity, primary language, type of issues raised at visit (e.g., hyperactivity, inattention, or disruptive behavior, depression, anxiety, eating issues, substance use/additional risk, trauma/violence, family stress and/or stress reaction, emergency services, chronic disease management, social/material needs, other BH concern, developmental concern, parent/caregiver mental health concern, early childhood concern, safety/suicidal ideation concern, and school-related concern), health-related social needs (e.g., needs for housing, food, transportation, and utilities), having other types of BH treatment, and time to follow-up screen after the index screen date.

© 2025 Kim J et al. *JAMA Network Open*.

eTable 6. Changes in PSC-17 item scores from baseline to follow-up by selected treatment and diagnosis type

PSC-17 item	Δ Mean (SD) [a]								
	Control	BHC encounter				Medication use			
	Total (Unique N=528)	Total (Unique N=368)	Anxiety/ Depression (Unique N=238)	Hyper-activity, inattention, or disruptive behavior (Unique N=136)	Emergency services (Unique N=74)	Total (Unique N=139)	Anxiety/ Depression (Unique N=69)	Hyperactivity, inattention, or disruptive behavior (Unique N=92)	Emergency services (Unique N=31)
Total	-0.34 (5.20)	-1.33 (6.34)	-1.12 (6.44)	-1.69 (6.79)	-1.44 (6.48)	-1.40 (5.41)	-1.96 (5.10)	-1.09 (5.49)	-2.39 (5.75)
Internalizing	-0.11 (2.11)	-0.53 (2.64)	-0.68 (2.79)	-0.19 (2.38)	-0.24 (2.55)	-0.22 (2.28)	-0.69 (2.33)	0.26 (2.10)	-0.09 (2.61)
Feels sad, unhappy	-0.02 (0.63)	-0.12 (0.72)	-0.14 (0.73)	-0.09 (0.71)	-0.06 (0.70)	-0.06 (0.68)	-0.14 (0.71)	0.05 (0.61)	-0.07 (0.67)
Feels hopeless	-0.05 (0.53)	-0.06 (0.68)	-0.10 (0.69)	-0.01 (0.60)	0.01 (0.63)	0.02 (0.67)	-0.07 (0.68)	0.11 (0.64)	0.06 (0.79)
Is down on him or herself	0.05 (0.61)	-0.08 (0.79)	-0.10 (0.83)	0.01 (0.77)	0.00 (0.87)	-0.02 (0.70)	-0.15 (0.71)	0.07 (0.64)	-0.04 (0.74)
Worries a lot	-0.02 (0.76)	-0.12 (0.85)	-0.18 (0.87)	0.00 (0.80)	-0.05 (0.88)	-0.13 (0.74)	-0.14 (0.72)	-0.08 (0.75)	0.07 (0.72)
Seems to be having less fun	-0.06 (0.70)	-0.17 (0.75)	-0.18 (0.78)	-0.10 (0.73)	-0.15 (0.71)	-0.02 (0.80)	-0.18 (0.77)	0.11 (0.78)	-0.10 (0.75)
Externalizing	-0.19 (2.38)	-0.60 (2.90)	-0.39 (2.62)	-1.00 (3.85)	-0.99 (2.94)	-0.81 (2.93)	-0.82 (2.36)	-0.93 (3.34)	-1.97 (3.65)
Fights with other children	-0.06 (0.57)	-0.08 (0.60)	-0.04 (0.55)	-0.22 (0.78)	-0.15 (0.59)	-0.15 (0.66)	-0.13 (0.54)	-0.20 (0.73)	-0.36 (0.76)
Does not listen to rules	-0.04 (0.67)	-0.14 (0.66)	-0.10 (0.62)	-0.25 (0.76)	-0.15 (0.56)	-0.19 (0.59)	-0.24 (0.52)	-0.16 (0.64)	-0.19 (0.51)
Does not understand other people's feelings	0.02 (0.66)	-0.06 (0.75)	-0.05 (0.77)	-0.08 (0.81)	-0.04 (0.60)	-0.09 (0.77)	-0.11 (0.66)	-0.09 (0.79)	-0.20 (0.77)
Teases others	0.00 (0.51)	-0.11 (0.58)	-0.08 (0.50)	-0.16 (0.74)	-0.23 (0.61)	-0.05 (0.66)	-0.01 (0.42)	-0.11 (0.78)	-0.36 (0.70)
Blames others for his or her trouble	0.01 (0.60)	-0.09 (0.64)	-0.04 (0.57)	-0.13 (0.83)	-0.21 (0.69)	-0.08 (0.65)	-0.04 (0.59)	-0.11 (0.71)	-0.38 (0.77)
Refuses to share	-0.06 (0.60)	-0.06 (0.69)	-0.04 (0.69)	-0.04 (0.81)	-0.12 (0.71)	-0.20 (0.72)	-0.23 (0.70)	-0.21 (0.74)	-0.33 (0.91)

© 2025 Kim J et al. *JAMA Network Open*.

Takes things that do not belong to him or her	-0.07 (0.49)	-0.05 (0.57)	-0.01 (0.54)	-0.09 (0.75)	-0.04 (0.60)	-0.08 (0.57)	-0.07 (0.54)	-0.09 (0.62)	-0.12 (0.83)
Attention	-0.01 (2.53)	-0.18 (2.75)	-0.06 (2.80)	-0.47 (2.64)	-0.21 (2.69)	-0.28 (2.42)	-0.34 (2.44)	-0.37 (2.31)	-0.21 (2.21)
Fidgety, unable to sit still	0.03 (0.83)	-0.05 (0.83)	0.03 (0.89)	-0.07 (0.77)	-0.14 (0.76)	-0.02 (0.79)	0.04 (0.81)	-0.06 (0.70)	0.03 (0.84)
Daydreams too much	-0.03 (0.78)	0.06 (0.85)	0.05 (0.90)	0.05 (0.76)	0.19 (0.86)	-0.04 (0.80)	-0.13 (0.75)	0.04 (0.84)	-0.06 (0.63)
Distracted easily	0.00 (0.76)	-0.06 (0.82)	-0.01 (0.84)	-0.16 (0.80)	-0.03 (0.78)	-0.06 (0.70)	-0.09 (0.74)	-0.12 (0.68)	0.02 (0.55)
Has trouble concentrating	-0.05 (0.80)	-0.08 (0.85)	-0.04 (0.79)	-0.20 (0.91)	-0.12 (0.90)	-0.11 (0.84)	-0.14 (0.78)	-0.14 (0.83)	-0.10 (0.82)
Acts as if driven by a motor	0.04 (0.75)	-0.07 (0.73)	-0.10 (0.69)	-0.09 (0.83)	-0.11 (0.63)	-0.06 (0.84)	-0.01 (0.69)	-0.10 (0.92)	-0.10 (0.82)

Note: [a] Δ Mean (SD) represents the change in score for each item (follow-up minus baseline). Negative values indicate symptom improvement. Anxiety/depression, hyperactivity/inattention/disruptive behavior, and emergency services were top three concerns/diagnoses raised by primary care providers among children who received each treatment.

© 2025 Kim J et al. *JAMA Network Open*.

eTable 7. Distribution of PSC-17 scores by behavioral health (BH) concern identification status

Identification status	Unique N (%)	Total score		Internalizing score		Externalizing score		Attention score	
		Mean (SD) [Median]	% with score ≥ 15	Mean (SD) [Median]	% with score ≥ 5	Mean (SD) [Median]	% with score ≥ 7	Mean (SD) [Median]	% with score ≥ 7
BH concern identified	6,973 (31%)	8.96 (5.95) [8]	19.2 %	2.75 (2.44) [2]	23.9 %	2.38 (2.60) [2]	8.4%	3.75 (2.74) [3]	18.8 %
BH concern not identified	15,488 (69%)	4.46 (4.27) [4]	2.7%	1.23 (1.59) [1]	5.9%	1.37 (1.84) [1]	1.9%	1.86 (2.00) [1]	3.0%
Total	22,461 (100%)	5.97 (5.33) [5]	8.18 %	1.74 (2.04) [1]	11.3 %	1.71 (2.17) [1]	4.1%	2.49 (2.45) [2]	8.3%

Note: This table represents distribution of PSC-17 scores among children identified vs. not identified as having a BH concern by their PCP. BH concern identification status was recorded in the Electronic Medical Record (EMR) based on the Primary Care Provider BH Plan—an EMR template completed after each visit to document identification of BH concerns and recommendations for BH services. Maximum value of the PSC-17 score is 34 for total score, 10 for internalizing and attention scores, and 14 for externalizing score. The PSC-17 cutoff for clinical referral is 15 for total score, 5 for internalizing score, and 7 for externalizing and attention scores. Out of 22,461unique children aged 4 to 18 years, 6,973 (31%) were identified by their PCP as having a BH concern (see Supplemental Figure 1).

Abbreviation: PSC-17=Pediatric Symptom Checklist (17-items); BH=behavioral health; SD=standard deviation.

© 2025 Kim J et al. *JAMA Network Open*.

eTable 8. Distribution of PSC-17 scores by type of behavioral health (BH) concern/diagnosis among children identified by their primary care provider as having a BH concern

Type of BH concern/ diagnosis	Unique N (%)	Total score		Internalizing score		Externalizing score		Attention score	
		Mean (SD)	% with score ≥ 15	Mean (SD)	% with score ≥ 5	Mean (SD)	% with score ≥ 7	Mean (SD)	% with score ≥ 7
Total	6,973 (100%)	8.96 (5.95)	19.2%	2.75 (2.44)	23.9%	2.38 (2.60)	8.4%	3.75 (2.74)	18.8%
Hyperactivity, inattention, or disruptive behavior	1,762 (25%)	11.11 (5.93)	28.8%	2.46 (2.18)	18.3%	3.47 (2.92)	15.9%	5.06 (2.71)	33.4%
Depression	1,824 (26%)	10.60 (5.64)	25.9%	4.38 (2.57)	49.1%	2.00 (2.29)	5.1%	4.17 (2.64)	21.6%
Anxiety	1,695 (24%)	9.75 (5.63)	22.1%	3.66 (2.41)	36.0%	1.97 (2.33)	5.6%	4.03 (2.74)	21.4%
Eating issues	191 (3%)	8.73 (5.52)	15.1%	2.98 (2.43)	26.9%	2.00 (2.29)	7.3%	3.68 (2.72)	16.4%
Substance use/addiction risk	114 (2%)	9.64 (5.84)	21.2%	3.26 (2.56)	35.4%	2.20 (2.51)	8.1%	4.15 (2.51)	21.2%
Trauma/violence	261 (4%)	9.50 (5.99)	19.2%	3.06 (2.38)	25.6%	2.39 (2.67)	10.2%	3.97 (2.78)	21.1%
Family stress and/or stress reaction	905 (13%)	9.93 (6.16)	23.7%	3.15 (2.45)	28.3%	2.74 (2.75)	12.0%	3.94 (2.77)	20.4%
Emergency services (section 12, ESP, DCF filing, etc.)	642 (9%)	9.05 (6.20)	21.1%	2.89 (2.41)	24.5%	2.46 (2.84)	9.4%	3.61 (2.74)	18.0%
Chronic disease management (medical)	169 (2%)	8.99 (5.72)	18.2%	2.32 (2.25)	17.0%	2.76 (2.72)	10.7%	3.81 (2.63)	15.7%
Social/material needs	870 (12%)	7.04 (5.75)	11.9%	1.88 (2.07)	12.2%	2.15 (2.50)	7.2%	2.93 (2.51)	9.9%
Other mental health concern	680 (10%)	8.34 (6.41)	19.7%	2.45 (2.37)	19.5%	2.51 (2.72)	9.0%	3.36 (2.85)	17.3%
Developmental concern	1,250 (18%)	9.26 (6.31)	21.9%	2.10 (2.19)	16.1%	3.07 (2.83)	13.1%	3.99 (2.83)	21.2%
Parent/caregiver mental health concern	1,304 (19%)	8.92 (6.10)	20.4%	2.46 (2.29)	20.1%	2.53 (2.68)	9.6%	3.79 (2.77)	19.9%
Early childhood concern (BRANCH)	19 (0%)	12.80 (6.50)	50.0%	2.38 (2.07)	12.5%	5.00 (3.67)	37.5%	5.25 (3.24)	37.5%
Safety/suicidal ideation concern	136 (2%)	12.51 (5.81)	42.8%	4.84 (2.77)	61.6%	2.72 (2.34)	6.5%	4.88 (2.69)	30.4%
School related concern	838 (12%)	9.94 (6.46)	24.8%	2.55 (2.40)	19.6%	3.00 (2.87)	12.1%	4.30 (2.84)	24.8%

Note: This table represents distribution of PSC-17 scores by type of behavioral health concern/diagnosis among children identified by their PCP as having a BH concern. Type of behavioral health (BH) concern/diagnosis was recorded in the Electronic Medical Record (EMR) based on the Primary Care Provider BH Plan—an EMR template completed after each visit to document identification of BH concerns and recommendations for BH services. Maximum value of the PSC-17 score is 34 for total

© 2025 Kim J et al. *JAMA Network Open*.

score, 10 for internalizing and attention scores, and 14 for externalizing score. The PSC-17 cutoff for clinical referral is 15 for total score, 5 for internalizing score, and 7 for externalizing and attention scores. Total number of unique children of 6,973 includes those aged 4 to 18 years who were identified by their PCP as having a BH concern (see Supplemental Figure 1).

Abbreviation: PSC-17=Pediatric Symptom Checklist (17-items); BH= behavioral health; SD=standard deviation.

© 2025 Kim J et al. *JAMA Network Open*.

eTable 9. Documented behavioral health concerns/diagnoses among children with non-elevated PSC-17 scores who were identified by their PCP as having a BH concern

Type of BH concern/ diagnosis	Unique N (%) of referred children with non-elevated PSC-17 score (<15)	Total score	Internalizing score		Externalizing score		Attention score	
		Mean (SD)	Mean (SD)	% with score ≥ 5	Mean (SD)	% with score ≥ 7	Mean (SD)	% with score ≥ 7
Total	4,903 (100%) [a]	6.81 (4.19)	2.15 (2.03)	14.9%	1.64 (1.86)	1.9%	2.97 (2.29)	8.4%
Hyperactivity, inattention, or disruptive behavior	1,152 (23%)	8.16 (3.92)	1.73 (1.72)	7.6%	2.28 (2.03)	3.1%	4.08 (2.39)	17.9%
Depression	1,176 (24%)	8.04 (3.88)	3.57 (2.21)	36.4%	1.26 (1.55)	0.6%	3.19 (2.12)	7.4%
Anxiety	1,061 (22%)	7.48 (3.92)	3.01 (2.06)	24.9%	1.27 (1.65)	1.2%	3.13 (2.25)	9.1%
Eating issues	121 (2%)	7.09 (4.15)	2.47 (2.04)	19.9%	1.47 (1.77)	2.2%	3.09 (2.42)	10.2%
Substance use/addiction risk	69 (1%)	7.37 (3.99)	2.47 (2.11)	20.5%	1.59 (2.05)	3.9%	3.30 (1.92)	6.4%
Trauma/violence	157 (3%)	7.30 (4.00)	2.48 (1.98)	16.7%	1.64 (1.84)	2.3%	3.13 (2.27)	9.8%
Family stress and/or stress reaction	527 (11%)	7.26 (4.03)	2.42 (2.03)	16.3%	1.80 (1.86)	2.4%	2.97 (2.22)	7.8%
Emergency services (section 12, ESP, DCF filing, etc.)	463 (9%)	6.60 (4.18)	2.27 (2.03)	14.5%	1.56 (1.88)	1.5%	2.72 (2.16)	6.3%
Chronic disease management (medical)	103 (2%)	7.09 (4.29)	1.92 (2.06)	12.3%	1.86 (1.80)	0.8%	3.23 (2.43)	11.5%
Social/material needs	632 (13%)	5.53 (4.13)	1.50 (1.76)	6.9%	1.58 (1.85)	1.9%	2.40 (2.07)	3.9%
Other mental health concern	409 (8%)	5.82 (4.08)	1.75 (1.77)	9.0%	1.65 (1.83)	1.3%	2.38 (2.11)	4.9%
Developmental concern	683 (14%)	6.72 (4.34)	1.45 (1.64)	6.5%	2.08 (2.02)	2.7%	3.12 (2.40)	9.5%
Parent/caregiver mental health concern	890 (18%)	6.57 (4.18)	1.86 (1.89)	11.2%	1.69 (1.91)	2.0%	2.95 (2.29)	8.5%
Early childhood concern (BRANCH)	3 (0%)	7.50 (4.80)	2.25 (0.96)	0.0%	2.25 (2.87)	0.0%	3.00 (2.16)	0.0%

© 2025 Kim J et al. *JAMA Network Open*.

Safety/suicidal ideation concern	60 (1%)	8.46 (3.91)	3.67 (2.64)	43.0%	1.60 (1.66)	1.3%	3.17 (1.86)	3.8%
School related concern	481 (10%)	7.07 (4.26)	1.80 (1.80)	9.3%	1.95 (1.98)	2.2%	3.29 (2.35)	10.8%

Note: This table represents number (%) of children with each behavioral health concern/diagnosis among those with non-elevated PSC-17 scores who were identified by their PCP as having a BH concern. Type of behavioral health (BH) concern/diagnosis was recorded in the Electronic Medical Record (EMR) based on the Primary Care Provider BH Plan—an EMR template completed after each visit to document identification of BH concerns and recommendations for BH services. Maximum value of the PSC-17 score is 34 for total score, 10 for internalizing and attention scores, and 14 for externalizing score. The PSC-17 cutoff for clinical referral is 15 for total score, 5 for internalizing score, and 7 for externalizing and attention scores.

[a] Total unique number of referred children with non-elevated PSC-17 score of 4,903 children includes those aged 4 to 18 years who were identified by their PCP as having a BH concern, with total PSC-17 score less than 15.

Abbreviation: PSC-17=Pediatric Symptom Checklist (17-items); SD=standard deviation.

© 2025 Kim J et al. *JAMA Network Open*.

eTable 10. Association between CHW encounter and the PSC-17 screen scores among children in TEAM UP FQHCs (a sensitivity analysis excluding data from Site 3)

	Coefficient[a] (95% CI[b])
Panel A. Primary regression analyses with an interaction term between treatment status & post status [c]	
Treatment	
Control group	(Reference)
Treated group	1.30 (0.07, 2.53)
Post	
Pre	(Reference)
Post	-0.97 (-3.41, 1.47)
Treatment×Post	
Control group×Post	(Reference)
Treated group×Post	-0.47 (-1.88, 0.94)
R^2	0.25
Number of Observation	1,314
Panel B. Secondary regression analyses with interaction terms between for the number of encounters & post status [c]	
# Encounters	
0 encounter	(Reference)
1 encounter	1.34 (-0.12, 2.80)
2 encounters	0.49 (-1.65, 2.63)
≥3 encounters	1.68 (-0.03, 3.39)
Post status	
Pre	(Reference)
Post	-0.76 (-3.24, 1.73)
# Encounters×Post	
0 encounter×Post	(Reference)
1encounter×Post	-0.55 (-2.25, 1.15)
2 encounters×Post	0.86 (-1.28, 3.01)
≥3 encounters×Post	-1.11 (-2.99, 0.77)
R^2	0.25
Number of Observation	1,314

[a] A negative coefficient of the interaction term means that the PSC score was lower (psychosocial functioning was improved) for patients with treatment after the treatment compared with patients without any treatment.
[b] 95% confidence interval (CI) in brackets.
[c] The model included year-specific effects, FQHC fixed effects, and a vector of patient-level covariates such as sex, age, race/ethnicity, primary language, type of issues raised at visit (e.g., hyperactivity, inattention, or disruptive behavior, depression, anxiety, eating issues, substance use/additional risk, trauma/violence, family stress and/or stress reaction, emergency services, chronic disease management, social/material needs, other BH concern, developmental concern, parent/caregiver mental health concern, early childhood concern, safety/suicidal ideation concern, and school-related concern), health-related social needs (e.g., needs for housing, food, transportation, and utilities), having other types of BH treatment, and time to follow-up screen after the index screen date.
Note: Given the small number of CHW encounters at Site 3, we conducted a sensitivity analysis excluding data from Site 3 (N=85).

© 2025 Kim J et al. *JAMA Network Open*.
