# Supplementary material for: Integrated Behavioral Health Services and Psychosocial Symptoms in Children
Source: JAMA Netw Open. 2025 Sep 16;8(9):e2532020. doi: 10.1001/jamanetworkopen.2025.32020 (PMC12441876; doi:10.1001/jamanetworkopen.2025.32020)
Supplement: Supplement 2. — Data Sharing Statement [file jamanetwopen-e2532020-s002.pdf]

Data Sharing Statement

Kim. Integrated Behavioral Health Services and Psychosocial Symptoms in Children. *JAMA Netw Open*. Published September 16, 2025. doi:10.1001/jamanetworkopen.2025.32020

Data
Data available: No
